# Taq-Polymerase Stop Assay to Determine Target Selectivity of G4 Ligands in Native Promoter Sequences of *MYC*, *TERT*, and *KIT* Oncogenes

**DOI:** 10.3390/ph16040544

**Published:** 2023-04-05

**Authors:** Galina V. Chashchina, Liana L. Tevonyan, Artemy D. Beniaminov, Dmitry N. Kaluzhny

**Affiliations:** 1 Engelhardt Institute of Molecular Biology, Russian Academy of Sciences, 119991 Moscow, Russia; 2 Moscow Institute of Physics and Technology, 141701 Dolgoprudny, Russia

**Keywords:** G4 structures, G4 ligands, TMPyP4, PDS, PhenDC3, BRACO-19, polymerase stop assay

## Abstract

Computational and high-throughput experimental methods predict thousands of potential quadruplex sequences (PQSs) in the human genome. Often these PQSs contain more than four G-runs, which introduce additional uncertainty into the conformational polymorphism of the G4 DNA. G4-specific ligands, which are currently being actively developed as potential anticancer agents or tools for studying G4 structures in genomes, may preferentially bind to specific G4 structures over the others that can be potentially formed in the extended G-rich genomic region. We propose a simple technique that identifies the sequences that tend to form G4 in the presence of potassium ions or a specific ligand. Thermostable DNA Taq-polymerase stop assay can detect the preferential position of the G4 –ligand binging within a long PQS-rich genomic DNA fragment. This technique was tested for four G4 binders PDS, PhenDC3, Braco-19, and TMPyP4 at three promoter sequences of *MYC*, *KIT*, and *TERT* that contain several PQSs each. We demonstrate that the intensity of polymerase pausing reveals the preferential binding of a ligand to particular G4 structures within the promoter. However, the strength of the polymerase stop at a specific site does not always correlate with the ligand-induced thermodynamic stabilization of the corresponding G4 structure.

## 1. Introduction

G-quadruplexes (G4s) are non-canonical secondary DNA structures that are formed by guanine-rich sequences. Four guanines form the G-quartet, a flat structure stabilized by Hoogsteen bonds. Potassium ions are known to stabilize stacking interaction between the G-quartets firmly. G4s participate in different biological processes, such as replication, transcription, translation, and others [1]. In the human genome, potential quadruplex sequences (PQSs) are spread in the promoters of oncogenes, 5’-untranslated regions, splicing sites, and telomers [2]. Since most human oncogene promoters contain PQSs, G4 structures are a promising therapeutic target for cancer treatment [3]. The search for G4 stabilizers was a general drive for developing G4-targeted low-molecular-weight compounds for anticancer therapy [4,5].

The presence of unique structural sites in the G4 DNA, such as the accessible guanine quartets, structured or single-stranded loops with exposed nucleotides or bulged-out bases, allows for specific recognition of G-quadruplexes by drugs and proteins [6]. Studies of such structural features on oligonucleotide models provide essential information for designing small molecules that interact with specific DNA sites in the genome. PQSs from promoter sequences can often form several interacting or overlapping G4 structures. Non-canonical polymorphism of G4s is involved in protein regulation cascades that differ between different G4 conformations [7]. The dynamic formation of G4 structures plays an important role in maintaining cell functions [8]. The complex topological landscape of different conformations of DNA with a local minimum allows PQSs to easily switch between G4 structures under the influence of various factors, such as local attenuation of the double helix, interaction with ligands, and addition of metal ions. Genomic regions contain sequences capable of forming more than one unique G4 structure, and interactions between them may additionally stabilize such structures. It was suggested that such extended sequences fold into more complex structures that can be studied in depth by structural and biophysical methods [9,10].

Traditionally, oligonucleotide sequences with a known 3D structure solved by NMR or X-ray methods are used for studying G4-ligand interactions. However, the short synthetic oligonucleotides do not provide sufficient information about the influence of genomic sequence context and the presence of the complementary strand on the formation of G4s in the cellular environment. Potential interactions between multiple G-quadruplexes in double/single-stranded DNA are also missed in the analysis of short G4-sequences, which is usually restricted to the study of the G4 stabilization effect by a ligand. Oncogene promoters frequently contain multiple overlapping PQSs. It is not obvious which of them would be preferentially folded in an extended genomic context and how a G4 ligand would influence this structural distribution. This knowledge is necessary to create the complete picture of the G4 dynamic folding in a long genomic DNA; the analysis of separate G4 fragments can never provide this information.

It is known that DNA-binding compounds can modify the structure making it more suitable for the ligand. The conformation preferences may shift stable equilibrium structures formed without the ligand in favor of another drug-stabilized structure. In addition, ligands prefer the interaction sites of G4 structures with the duplex [11], and by analogy, one can assume the possibility of preferential stabilization of the G4-G4 junction. These junctions are potential ligand-binding sites, but it is unclear which one would be selected by the drug, and this information might be important for further detailed structural characterization of the target. The development of new approaches to the analysis of the preferred drug binding site on DNA stabilizing certain structures is relevant.

**Figure 1:**
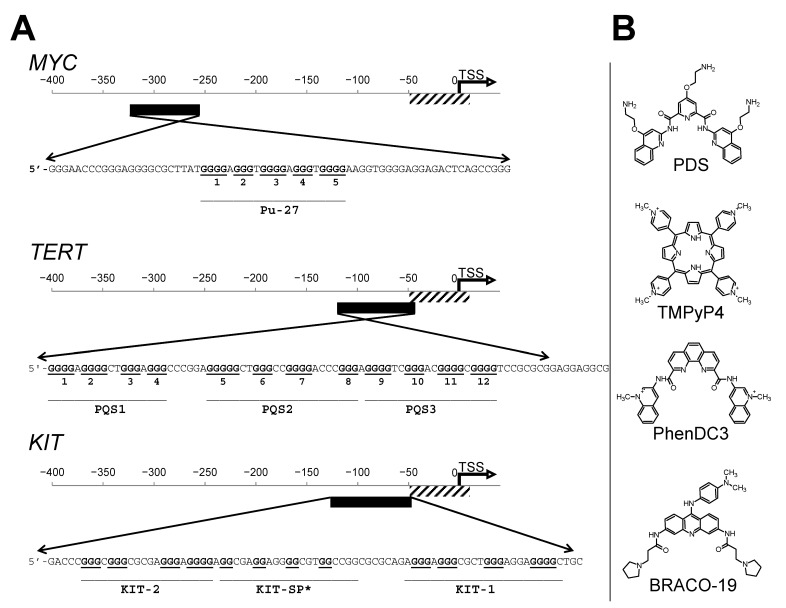
(**A**) Interplay of G4 in PQS in promoter regions of a genes *MYC, TERT,* and *KIT*. G-runs involved in canonical G4 structures marked with bold and underlined. The names of the canonical G4 structure are under the line that combines the G-runs. TSS corresponds to an alternative transcription start site from EPDnew [21]. (**B**) Schematic representation of compounds used as a tested G4 ligands.

We selected three long (~400 b.p.) human oncogene promoter regions of *MYC*, *KIT*, and *TERT*, which contain several overlapping PQSs (Figure 1A). *MYC* oncogene overexpression is associated with a wide range of human cancers, including carcinomas of the breast and lungs, osteosarcoma, lymphoma, leukemia, and others [12]. One strategy in inhibition of the *MYC* gene expression is to stabilize G4 structures in its promoter [13]. Various ligands targeting G4 have been tested to regulate *MYC* expression, some of which led to a decrease in tumor growth, which correlated with a decrease in *MYC* and other oncogenes expression [14]. Almost 90% of the *MYC* transcription is controlled by the region NHE III_1_, whose 27 nucleotide G-rich site pu27 consists of five consecutive runs of guanines (1, 2, 3, 4, and 5). Dimethylsulphate (DMS) footprinting showed that, in the presence of K^+^, the pu27 formed a quadruplex of the guanine runs 2, 3, 4, and 5 [15]. However, a recent footprinting study showed that in a supercoiled plasmid, the G4 was formed from 1, 2, 3, and 4 runs of guanines [16].

Human telomerase reverse transcriptase (hTERT) is the catalytic subunit of telomerase, an enzyme mainly responsible for the immortality of cancer cells. Telomerase activity is usually undetectable in somatic cells (except for stem cells), and its overexpression is associated with cancer. The almost exclusive expression of *TERT* in cancer cells is recognized as a target for anticancer therapy [17]. The *TERT* promoter region contains twelve runs of three or more guanines, which can give rise to three G4 structures (PQS1, PQS2, and PQS3). The secondary structure of the *TERT* promoter was intensely studied. The model proposed by Palumbo et al., supported by DMS modification results, showed a parallel PQS1 stacked onto an antiparallel/hybrid G-quadruplex with 8-bp hairpin loop [18]. Micheli et al. proposed another model that includes three adjacent parallel quadruplexes, which is explained by the stabilization of PQS2 through terminal G-quadruplexes [19]. Later, NMR studies found that the *TERT* promoter sequence folds into a compact stacked three G-quadruplex conformation [20].

Oncogene *KIT* encodes a tyrosine kinase receptor, which is predominantly expressed in mast cells, melanocytes, and hematopoietic stem cells. Overexpression and/or mutation of *KIT* can play a role in some gastrointestinal tumors, acute myeloid leukemia, and other cancers [22]. The *KIT* promoter contains three PQSs in a relatively long sequence potentially capable of forming several adjacent G4 structures: KIT-1, the Sp1 binding site—KIT-SP*, and KIT-2. PQSs KIT-2 and KIT-1 are folded into parallel quadruplexes. The KIT-SP* PQS contributes to the G4 structure only together with KIT-2 [23]. It is still not clear which one of the three PQSs is more biologically significant. The numbers of drug candidates for one or another G4 structure in the *KIT* promoter have been synthesized [24]. Interactions between potential drugs and G4 DNA have mainly been studied on individual independent quadruplex units derived from short truncated sequences without regard to the polynucleotide neighboring [25].

This work focuses on a biochemical approach for precise G4 detection in the extended G-rich sequence. The approach is based on Taq DNA polymerase inhibition by specific ligands in a normal PCR reaction and subsequent mapping of the stops in the gel. For the proof of concept, we chose four G4 ligands (Figure 1B): TMPyP4 [26], PDS [27], PhenDC3 [28], and BRACO-19 [29] and two double-strand binders Actinomycin D and Doxorubicin that served as negative controls.

The pattern of polymerase pause sites is specific to a G4 ligand and differs from potassium-induced pauses. Although Förster resonance energy transfer (FRET) melting confirms the high-affinity binding of a ligand to certain G4 structures, it cannot point to the preferable G4 targets out of many possible. We demonstrate that polymerase pause assay assists in the selection of compounds that bind to specific DNA sites.

## 2. Results and Discussion

### 2.1. Pause Sites of the Thermostable DNA Polymerase Are Caused by the Stabilization of G4 Structures by DNA-binding Ligands

**Figure 2:**
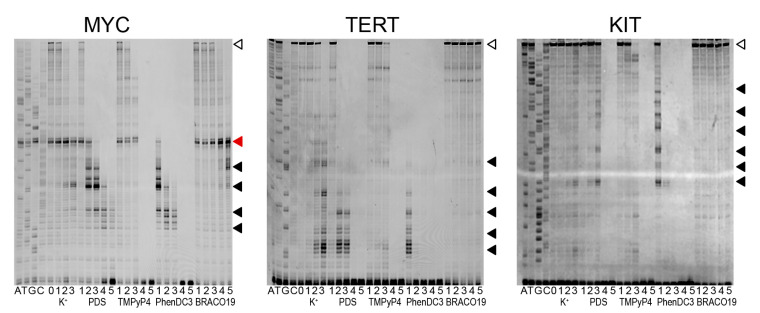
Products of elongation of a FAM-labeled primer on quadruplex sequences in the promoter regions of human genes (*MYC*, *KIT* and *TERT*) in the presence of PDS, TMPyP4, PhenDC3, BRACO-19, and potassium ions in different concentrations. The profile was obtained in a denaturing 10% PAAG. Lanes 0–3 for K^+^ corresponds to potassium concentration of 0, 1, 5, 10 mM in the primer extension reaction. Lanes 1–5 for compounds are for concentrations 0.1, 0.5, 1, 5, 10 µM, respectively. Polymerase pauses were marked by black-filled triangles. The red triangle indicates a non-G4 type of polymerase pausing. The open triangle is a full-length product.

Polymerase stop assay is one of the techniques used for detecting G4 structures in DNA sequences. It is based on the fact that stable secondary structures in a single-stranded template become an obstacle to polymerase synthesizing the complementary DNA strand [30]. This method is also used for testing potential G4 ligands [31]. Here, we used double-stranded DNA promoter regions of the human genes *MYC*, *KIT*, and *TERT* obtained by PCR amplification of the genomic DNA. The amplicons had an average length of 400 bp and contained several overlapping PQSs. The ability to induce polymerase pausing in PCR in the presence of potassium ions was demonstrated earlier [32]. The advantage of using elongated double-stranded DNA templates over synthetic oligonucleotides is the choice of a longer strand, making it possible to analyse structures in a natural genomic context. We selected primers that bind next to the multiple potential G4 sites. Polymerase pause sites were detected in denaturing PAGE and assigned using Sanger sequencing run in parallel. Primer extension assay was performed in the presence of quadruplex ligands PDS, TMPyP4, PhenDC3, BRACO-19, and potassium ions (Figure 2).

In the absence of any stabilizer (lane 0) or in the presence of low ligands concentration (100 nM), the polymerase reaches the end of the matrix, and we see a full-length PCR product marked with an open triangle in Figure 2 (except PhenDC3). At higher concentrations, the full-length product is absent, which indicates the inhibition of the polymerase by the compounds, and this effect is concentration-dependent.

PDS and PhenDC3 ligands and potassium ions caused concentration-dependent pause sites (black triangles), that may be associated with the stabilization of G4 structures on the DNA matrix strand. The most intensive pauses were observed for *MYC* and *TERT* templates. Compared with potassium, pausing sites with ligands appeared in different places, indicating that ligands bind to alternative G4 structures. For the *KIT* sample, pauses with PDS and PhenDC3 were visible at high concentrations only. The assignment of pause sites to specific nucleotide sequences is analyzed below. A strong polymerase pause (red triangle) was observed before a potential hairpin structure for the *MYC* promoter sequence. The intensity of this pause was not sensitive to K^+^ or G4-specific ligands and thus omitted.

Polymerase pauses were not detected for TMPyP4 and BRACO-19 compounds or were poorly defined. This result may indicate their low selectivity to G4 structures or insignificant stabilization of the G4 structures. The pause sites induced by BRACO-19 were seen only for *MYC* at high concentrations (5 µM) of the ligand.

Ligand binding to specific sites within the chosen promoter sequences can be derived from polymerase pausing sites between the position of the primer and full-length product and can be determined with nucleotide resolution.

As a control we used the well-known double-stranded DNA ligands doxorubicin (Dox) and actinomycin D (ActD) (Figure S1). Both ActD and Dox do not cause polymerase pause sites specific for G4 sequences. Nonetheless, strong binding of these ligands to G4 has previously been shown. For the *MYC* promoter, it was shown that ActD binds to double-stranded DNA with higher affinity than to the G4 structure [33]. Dox showed a strong and selective association with the VEGF Pu22 G4 structure, which was comparable to its well-known association with dsDNA [34]. Here we demonstrated that the polymerase stop assay was able to distinguish between poor and strong G4 binders.

### 2.2. Polymerase Pause Sites Correspond to G4 Sequences

**Figure 3:**
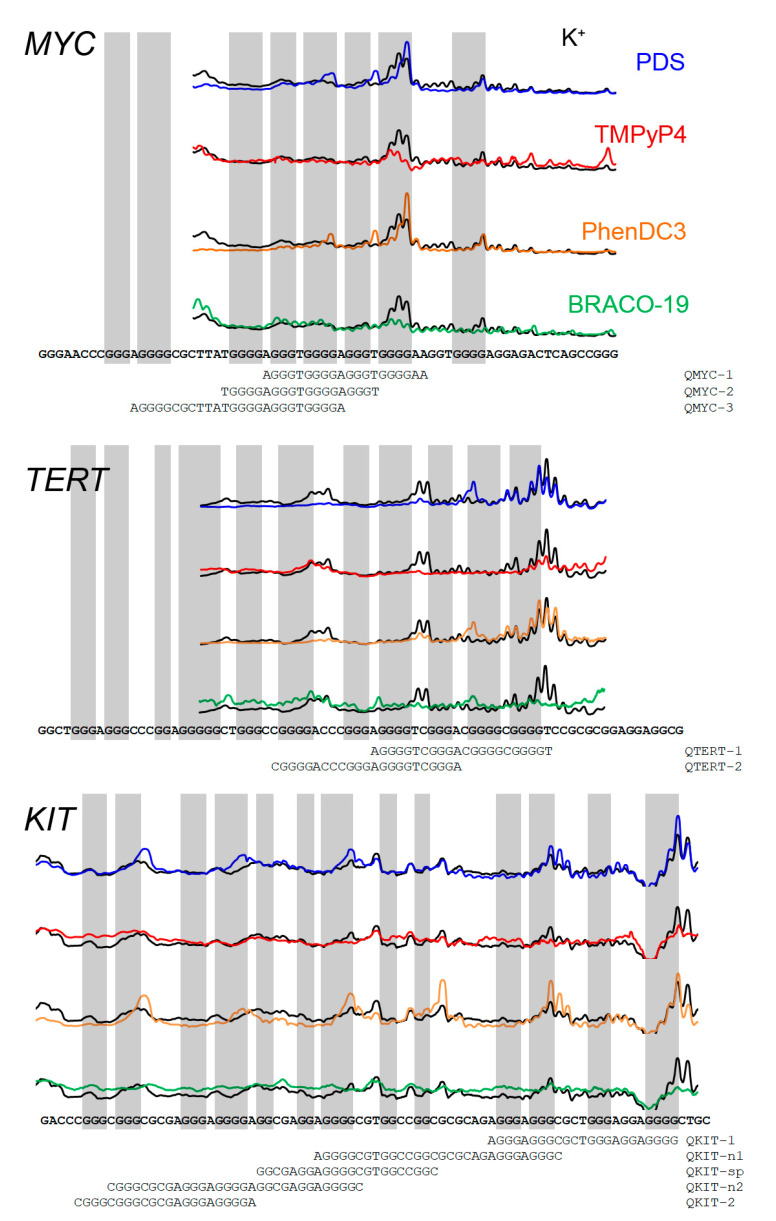
Densitometric profiles of primer extension lanes in the presence of G4-stabilizer in Taq-polymerase elongation reaction. Black—10 mM KCl, blue—0.5 µM PDS, red 1 µM—TMPyP4, orange—0.1 µM PhenDC3, green 5 µM BRACO-19. Oligonucleotides for FRET melting assay are shown under the promoter sequences.

DNA polymerase pause sites were correlated with the position of quadruplex sequences (Figure 3).

The polymerase pause sites correspond to the 3’-end of the sequence capable of forming a G-structure. We observed that long G-rich sequences could provide several bands of different intensities that may result from different stabilization effects for different G4 structures. To test this hypothesis, we synthesized several model oligonucleotides, the 3’ ends of which matched with the DNA polymerase pause sites (Figure 3). Several overlapping G4 sequences were identified for the *MYC* sequence. Pause sites with potassium ions or PhenDC3 and PDS ligands occur predominantly at the 3′ end of the Pu-27 sequence on the fifth guanine run. Three DNA oligonucleotides from the *MYC* promoter sequence were selected for future stability analysis. Sequence QMYC-1 corresponds to the most effective stop both with potassium and ligands. QMYC-2 and QMYC-3 are for stops observed only for PDS and PhenDC3 compounds.

For the *TERT* promoter region, polymerase pause sites in the presence of potassium ions coincide with the 3’ end of PQS2 and PQS3 (names from Figure 1). In the presence of PhenDC3 and PDS ligands, pause sites are seen only on the PQS3 sequence and additional new one stop observed inside PQS3. Pause sites at the PQS1 sequence were not observed, possibly because the polymerase did not reach this sequence and stopped earlier. We choose two DNA oligonucleotides: QTERT-1 for canonical PQS3 and QTERT-2 for a stop observed with PhenDC3 and PDS ligands.

For the *KIT* sequence, polymerase pause sites corresponded to three known quadruplexes described in the literature (KIT-1, KIT-SP*, KIT-2). On KIT-2, the pauses were not clearly visible, probably because the sequence was too long and the polymerase interrupted the synthesis earlier. Five DNA oligonucleotides were used for stability analysis: three of them were for canonical structures (named QKIT-1, QKIT-sp, QKIT-2) and two others that have not previously been explored, QKIT-n1 and QKIT-n2.

### 2.3. FRET Melting Assay of DNA Sequences Corresponded to Polymerase Pause Sites

**Figure 4:**
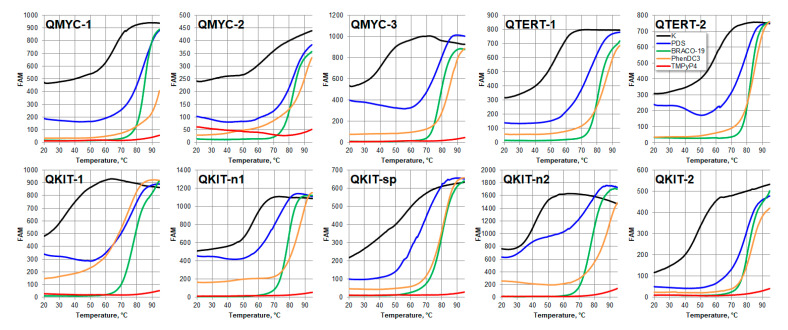
FRET melting profiles for DNA oligonucleotides corresponded with the polymerase pause sites in the presence of potassium ions or G4 ligands (10 mM KCl (black), 0.5 µM PDS (blue), 2.5 µM BRACO-19 (green), 0.5 µM PhenDC3 (orange) and 1 µM TMPyP4 (red)).

The stability of single-stranded DNA oligonucleotides was tested by a FRET melting assay [35]. Double-labeled oligonucleotides with fluorescein (FAM) at the 5′ end and carboxytetramethylrhodamine (TAMRA) at the 3′ end were melted in the presence of potassium or the G4 ligands. Melting profiles were registered by FAM fluorescence (Figure 4). Melting points were estimated as a maximum of the first derivative of the melting curves. The stabilities of the oligonucleotides are summarized in Table 1.

We found a lack of a strict correlation between Taq polymerase stops in the long promoter sequence and the stability of oligonucleotide FRET models. Both potassium- and ligand-induced stabilization is ambiguously correlated with the capacity of the ligands to induce polymerase stops. Most probably, the short DNA fragments do not fully simulate the stability of secondary structures in the context of an extended promoter sequence. Additional interactions with neighboring G4 structures or single-stranded or structured flanks may affect the stability of the structure in an extended context.

The stability of folded DNA induced by PDS and PhenDC3 is much higher than with potassium ions. Thus the folded single stranded sequences in complex with PDS or PhenDC3 can be a considerable obstacle for polymerase elongation in a PCR-stop assay. Interesting results were obtained for the TMPyP4 and BRACO-19 compounds, which showed little selectivity in primer polymerase elongation. It was shown that oligonucleotides in complexes with these compounds also exhibited high thermal stability. For TMPyP4, the denaturation temperatures of the complex could not be reached for all of the sequences studied. Thus, steady-state thermal stability does not fully describe the ability of compounds to pause polymerase elongation in certain regions of the DNA matrix. As a possible explanation of this phenomenon, it can be assumed that an important factor in the interaction of ligands with DNA is their kinetics of binding and dissociation. Previously, we have shown that the kinetics of dissociation of Olivomycin A from DNA is a factor in inhibiting RNA polymerase at certain sites of double-stranded DNA templates [36]. On the other hand, the presence of a complementary strand and a non-G4 flanking sequence makes the presented assay preferable in search of G4 ligands specific to a particular region of the genome.

## 3. Materials and Methods

### 3.1. Reagents

All chemicals, including TMPyP4, Meso-Tetra (N-methyl-4-pyridyl)porphine); PDS or Pyridostatin, 4-(2-Aminoethoxy)-N2,N6-bis(4-(2-aminoethoxy)quinolin-2-yl)pyridine-2,6-dicarboxamide; PhenDC3, 3,3′-[1,10-Phenanthroline-2,9-diylbis(carbonylimino)] bis[1-methylquinolinium]; BRACO-19, N,N′-(9-(4-(Dimethylamino)phenylamino) acridine-3,6-diyl)bis(3-(pyrrolidin-1-yl)propanamide); Actinomycin D 2-amino-4,6-dimethyl-3-oxo-1-N,9-N-bis[7,11,14-trimethyl-2,5,9,12,15-pentaoxo-3,10-di(propan-2-yl)-8-oxa-1,4,11,14-tetrazabicyclo[14.3.0]nonadecan-6-yl]phenoxazine-1,9-dicarboxamide); Doxorubicin (7S,9S)-7-[(2R,4S,5S,6S)-4-amino-5-hydroxy-6-methyloxan-2-yl]oxy-6,9,11-trihydroxy-9-(2-hydroxyacetyl)-4-methoxy-8,10-dihydro-7H-tetracene-5,12-dione) were purchased from Sigma Aldrich Chemicals (St. Louis, MO, USA), dissolved in DMSO to 10 mM concentration stored at 4 °C as stock solutions until further use.

### 3.2. Oligonucleotides

Primers for PCR were synthesized in Evrogen (Moscow, Russia). Fluorescently 5′-FAM labeled primers and double labeled 5′-FAM–3′-TAMRA oligonucleotides for FRET melting assay were synthesized by DNA Synthesis (Moscow, Russia). The sequences are given in Table 2.

### 3.3. Amplification of Promoter Regions

PCR was performed in 25-μL reaction containing 20 mM Tris-HCl, pH 9.5, 20 mM CsCl, 2.5 mM MgCl_2_, 5% dimethyl sulfoxide (DMSO), 0.25 μM dNTP, 0.5 μM each primer, 1U Encyclo DNA polymerase (Evrogen, Moscow, Russia), and 10 ng genomic DNA Raji (Evrogen, Moscow, Russia) was added as a template. PCR was carried out as described above under the following conditions: 95 °C for 3 min (1 cycle), 95 °C for 30 s, 60 °C for 30 s, 72 °C for 30 s (30 cycles), and final extension at 72 °C for 3 min. Amplicons were separated in 1% agarose gel with subsequent staining with ethidium bromide (1.5 µg/mL) and analyzed in UV light with GelDocTM XR+ transilluminator (Bio-Rad, Hercules, CA, USA).

### 3.4. Primer Extension in the Presence of Ligands

Reactions were performed in 25 μM in PCR buffer with 0.1 μM FAM-labeled primer, 0.1 mM dNTP, 0.5U SNP polymerase (Evrogen, Moscow, Russia), and 2 nM amplicons used as a template. Ligands of various concentrations (from 0.1 to 10 μM) or KCl of (1, 5, 10 mM) were added to the reaction mixture. For Sanger sequencing, deoxynucleotides ddA, ddT, ddG, or ddC were added to the concentrations 0.1, 0.1, 0.1, and 0.01 μM, respectively. The letters in the sequencing lanes corresponded to the deoxynucleotides added to the reaction mixture.

Primer extension was carried out as described above under the following conditions: 95 °C for 3 min (1 cycle), 95 °C for 30 s, extension for 30 s, 72 °C for 30 s (20 cycles), and the final extension at 72 °C for 3 min. The hybridization temperatures for primers were initially chosen using the program OligoAnalyzer Tool on the website idtdna.com (© 2023 Integrated DNA Technologies, Inc., Coralville, IA, USA) and corrected empirically.

The samples were precipitated by adding 200 μL 0.3 M NaAc, pH 5.3, and 600 μL 96% EtOH and centrifuged for 10 min at maximum speed (14,000× *g*). After washing with 70% EtOH, samples were dissolved in a loading buffer containing 30 mM Na-phosphate buffer, pH 7.8, 80% formamide, 0.025% Bromophenol blue, and 0.025% Xylene cyanol. Samples were denatured for 5 min at 95 °C and loaded in 10% polyacrylamide gel (acrylamide:bis-acrylamide, 19:1) containing 7 M urea and 1× TBE. Electrophoretic bands were visualized by scanning on Typhoon 9500 fluorescent image scanner (GE Healthcare, Chicago, IL, USA).

Densitometric profiles of the lanes corresponding to 10 mM KCl, 0.5 µM PDS, 1 µM TMPyP4, 0.1 μM PhenDC3 and 5 µM BRACO-19 were analyzed with ImageJ software and linearized by sequencing lanes for ddT or ddG.

### 3.5. FRET Melting Assay

Double-labeled DNA oligonucleotides with FAM at 5′ end and TAMRA at the 3′ end (sequences shown on Figure 3). The oligonucleotides were annealed in PCR buffer by heating to 95 °C for 5 min and then cooled to 20 °C for 15 min. The ligands’ concentrations were 10 mM KCl, 0.5 µM PDS, 1 µM TMPyP4, 0.5 µM PhenDC3, and 2.5 µM BRACO-19. The temperature dependence of FAM fluorescence was recorded using Applied Biosystems QuantStudio 5 Real-Time PCR System. The excitation of the fluorescence was at 470 ± 15 nm with fluorescence detection at 520 ± 15 nm. The melting procedure started with a 1 min incubation at 20 °C and followed by a temperature ramp at a 1 °C/min rate with fluorescence detection each 0.2 °C up to 95 °C. The melting point was estimated as a maximum of the first derivative of the melting curve registered with FAM fluorescence. To obtain a first derivative, the ratio of fluorescence increment to the temperature step of 1 °C was calculated. All melting curves are obtained in duplicate.

## 4. Conclusions

This work demonstrates that a simple biochemical approach can be used to establish the preference for compound binding to G4 DNA structures on extended natural nucleotide sequence templates. Four G4-ligands (PDS, PhenDC3, TMPyP4 and BRACO-19) were tested on three (*MYC*, *KIT*, *TERT*) natural promoters of double-stranded templates that contained multiple G4 structures. With single-nucleotide resolution, the method can determine the specific G4 structures the ligand preferentially interacts with when it has multiple G4 target variants. We showed the differential pausing abilities of potassium ions and the ligands to stop polymerase elongation. The lack of a direct correlation between the ability of potassium or the ligands to stop the polymerase and thermodynamic stabilization of the G4 structures may reflect the effect of the nearby matrix sequences in the extended promoter DNA templates.

The proposed approach can potentially be extended to screening a larger number of small molecules, including novel inducers and stabilizers of G4 structures. Although we limited the analysis to only three promoter sites, the same approach could be applied to other genomic regions of interest containing multiple G-runs. High-throughput genome-wide analysis of the compound specificity could be complicated by special conditions in the presence of compounds, but we believe it is still feasible. Nevertheless, the approach has been validated at three specific genomic sites and opens perspectives for large-scale specificity analysis. The results of this work confirm once again the need for a deeper understanding of G4/ligands’ interactions and for a validation of the current techniques. New tools are required to measure the effect of G4 ligands on extended genomic regions that would assist in the selection of more reliable G4 DNA targets for potential forthcoming drugs.

## Figures and Tables

**Table 1:** Stability of folded DNA structures obtained by FRET melting assay in presence of potassium or G4 ligands.

Oligonucleotide Name	KCl	PDS	TMPyP4	PhenDC3	BRACO-19
QMYC-1	66.4 ± 1.4	83.8 ± 1.1	>95	>95	85.4 ± 0.3
QMYC-2	66.4 ± 1.1	81.1 ± 3.8	>95	91.3 ± 1.6	82.1 ± 0.4
QMYC-3	44 ± 3.1	80.3 ± 1.8	>95	86.2 ± 0.3	78.3 ± 0.7
QTERT-1	55.7 ± 1.8	74.3 ± 1.3	ND	86.4 ± 0	81 ± 0.6
QTERT-2	58.6 ± 0.8	79.6 ± 0.3	ND	85.4 ± 0	82.2 ± 0.6
QKIT-1	33.6 ± 0.8	75 ± 2.8	>95	74.1 ± 3.3	77.9 ± 0.1
QKIT-n1	56.7 ± 0.4	68.2 ± 2	>95	87.4 ± 1.4	79.1 ± 0.1
QKIT-sp	56.3 ± 1	68.7 ± 1.6	>95	81.4 ± 0.3	75.6 ± 6.5
QKIT-n2	41.1 ± 0.4	79.8 ± 0.8	>95	89 ± 0	77 ± 0.8
QKIT-2	48.3 ± 1.3	78.3 ± 1.8	>95	83.9 ± 0.7	79.7 ± 0.1

**Table 2:** Primers and oligonucleotides used for analysis.

	Primers for PCR	Fluorescently Labeled Primers for Localizing DNA Polymerase Pause Sites
*MYC*	For: GAGGAGCAGCAGAGAAAGGG Rev: TCCCTCCGTTCTTTTTCCCG	FAM-TCCTAGAGCTAGAGTGCTCGG
*KIT*	For: GTGGAGAGAGAAAGGGGCTC Rev: AAGCAGTAGGAGCAGAACGC	FAM-GCGGCAAAGCCGAGCC
*TERT*	For: GGCCGATTCGACCTCTCT Rev: CAGCGCTGCCTGAAACTC	FAM-CTTCCAGCTCCGCCTCCTCC

## Data Availability

Data is contained within the article and supplementary material.

## Supplementary Materials

The following supporting information can be downloaded at: https://www.mdpi.com/article/10.3390/ph16040544/s1, Figure S1: (A) Products of elongation of a FAM-labeled primer in the promoter regions of human genes (*MYC, KIT,* and *TERT*) in the presence of potassium ions, Actinomycin D, and Doxorubicin in different concentrations (0-10 µM). The products were separated in a denaturing 10% PAAG. Concentration of KCL is given in mM. Concentration Actinomycin D, and Doxorubicin is in µM. Black triangles indicate polymerase pauses. The red triangle indicates the supposed hairpin. The open triangle is a full-length product. (B) Chemical structures of Doxorubicin and Actinomycin D; Figure S2: The first derivative plots of FRET melting profiles for DNA oligonucleotides corresponded the polymerase pause sites in presence of potassium ions or G4 ligands.
