# Supplementary material for: Taq-Polymerase Stop Assay to Determine Target Selectivity of G4 Ligands in Native Promoter Sequences of MYC, TERT, and KIT Oncogenes
Source: Pharmaceuticals (Basel). 2023 Apr 5;16(4):544. doi: 10.3390/ph16040544 (PMC10142109; doi:10.3390/ph16040544)
Supplement: Supplementary file 1 [file pharmaceuticals-16-00544-s001.zip › pharmaceuticals-2258734-supplementary.pdf]

Supplementary material

Taq-polymerase stop assay to determine target selectivity of G4 ligands in native promoter sequences of MYC, TERT, and KIT oncogenes

G.V. Chashchina, L.L. Tevonyan, A.D. Beniaminov, D.N. Kaluzhny*.

*uzhny@mail.ru

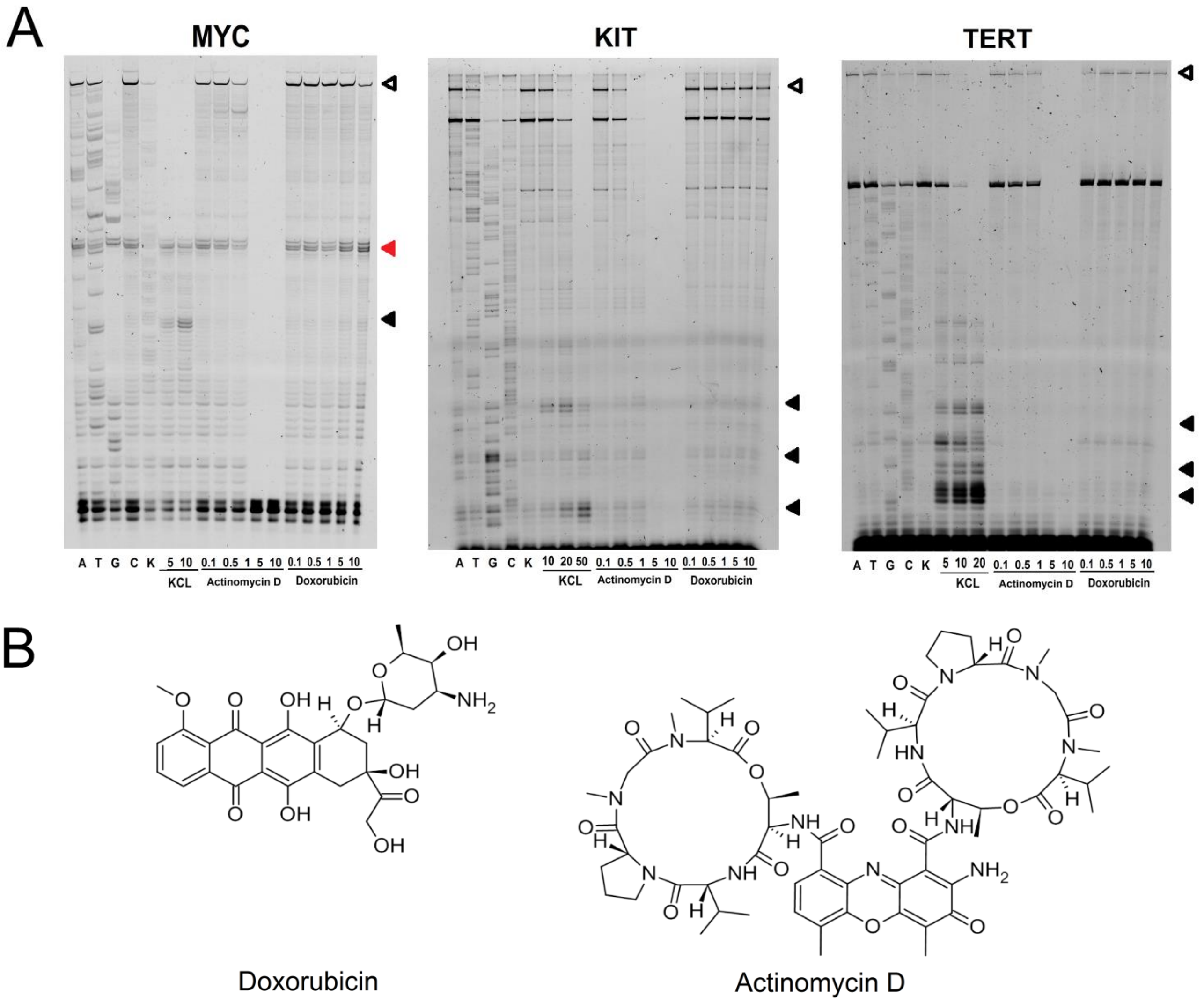

Figure S1. (A) Products of elongation of a FAM-labeled primer in the promoter regions of human genes (*MYC*, *KIT*, and *TERT*) in the presence of potassium ions, Actinomycin D, and Doxorubicin in different concentrations (0-10 μM). The the products were separated in a denaturing 10% PAAG. Concentration of KCL is given in mM. Concentration Actinomycin D, and Doxorubicin is in μM. Black triangles indicate polymerase pauses. The red triangle indicates the supposed hairpin. The open triangle is a full-length product. **(B)** Chemical structures of Doxorubicin and Actinomycin D.

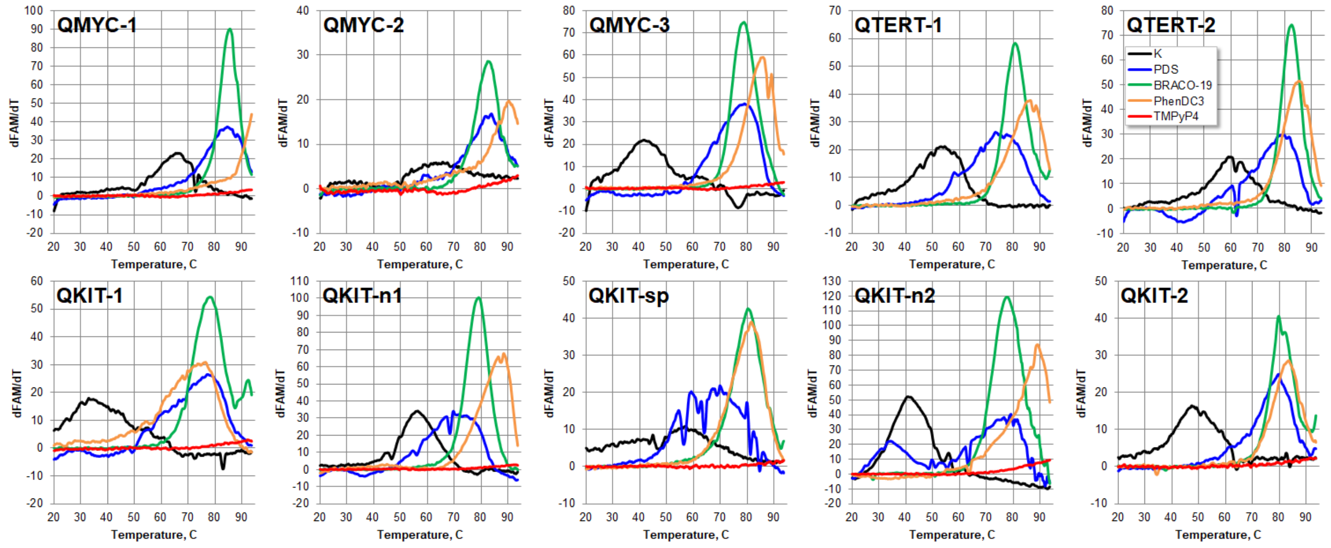

Figure S2. The first derivative plots of FRET melting profiles for DNA oligonucleotides corresponded the polymerase pause sites in presence of potassium ions or G4 ligands.
